# Supplementary figures and images for: Characteristics of the gut microbiome in patients with prediabetes and type 2 diabetes
Source: PeerJ. 2021 Mar 24;9:e10952. doi: 10.7717/peerj.10952 (PMC8000457; doi:10.7717/peerj.10952)

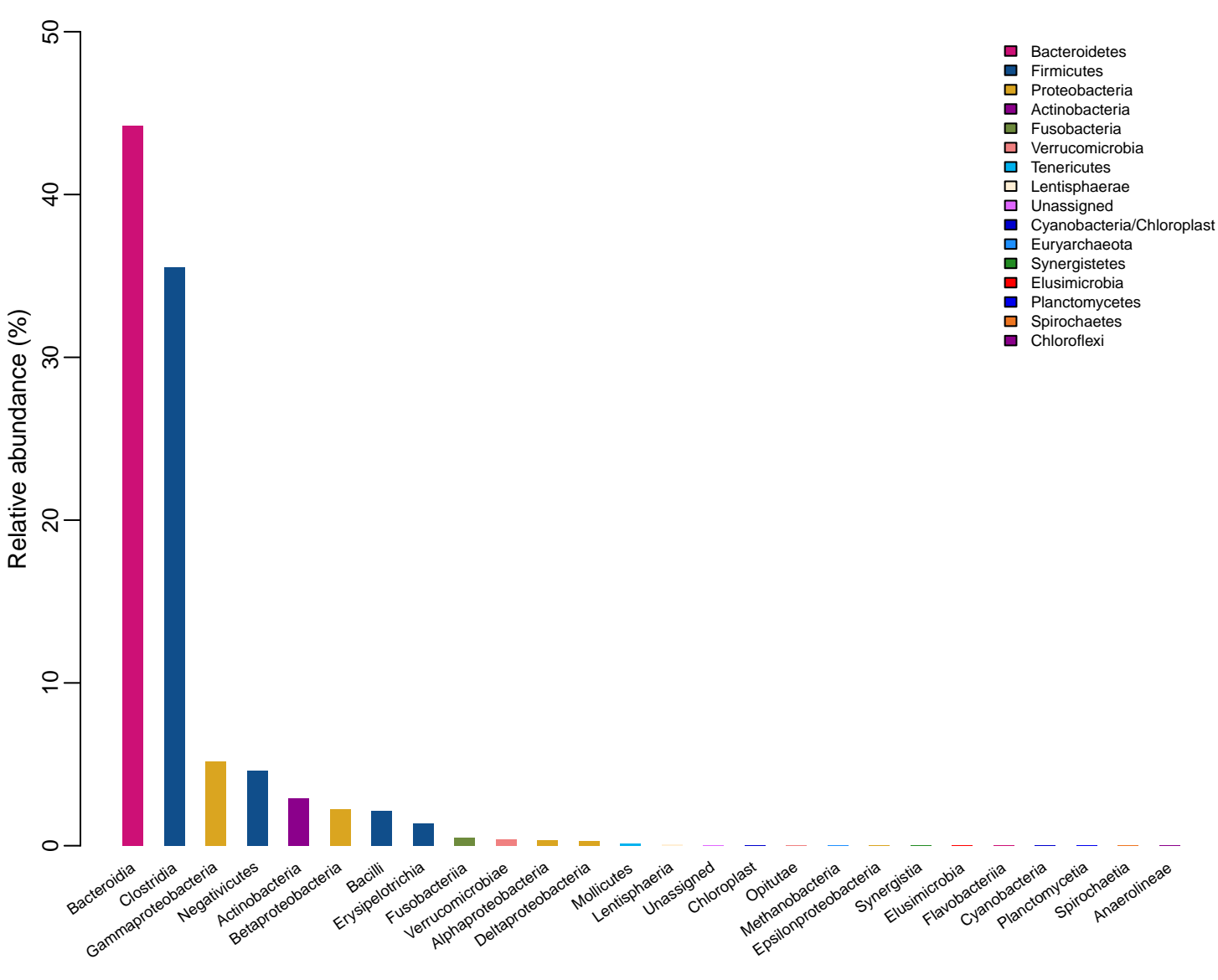

Supplement: Supplemental Information 1 [file peerj-09-10952-s001.zip › data/group_result_1_2_3/Community_Structure/class/class.taxon.Barplot.Legend.With.phylum.pdf]

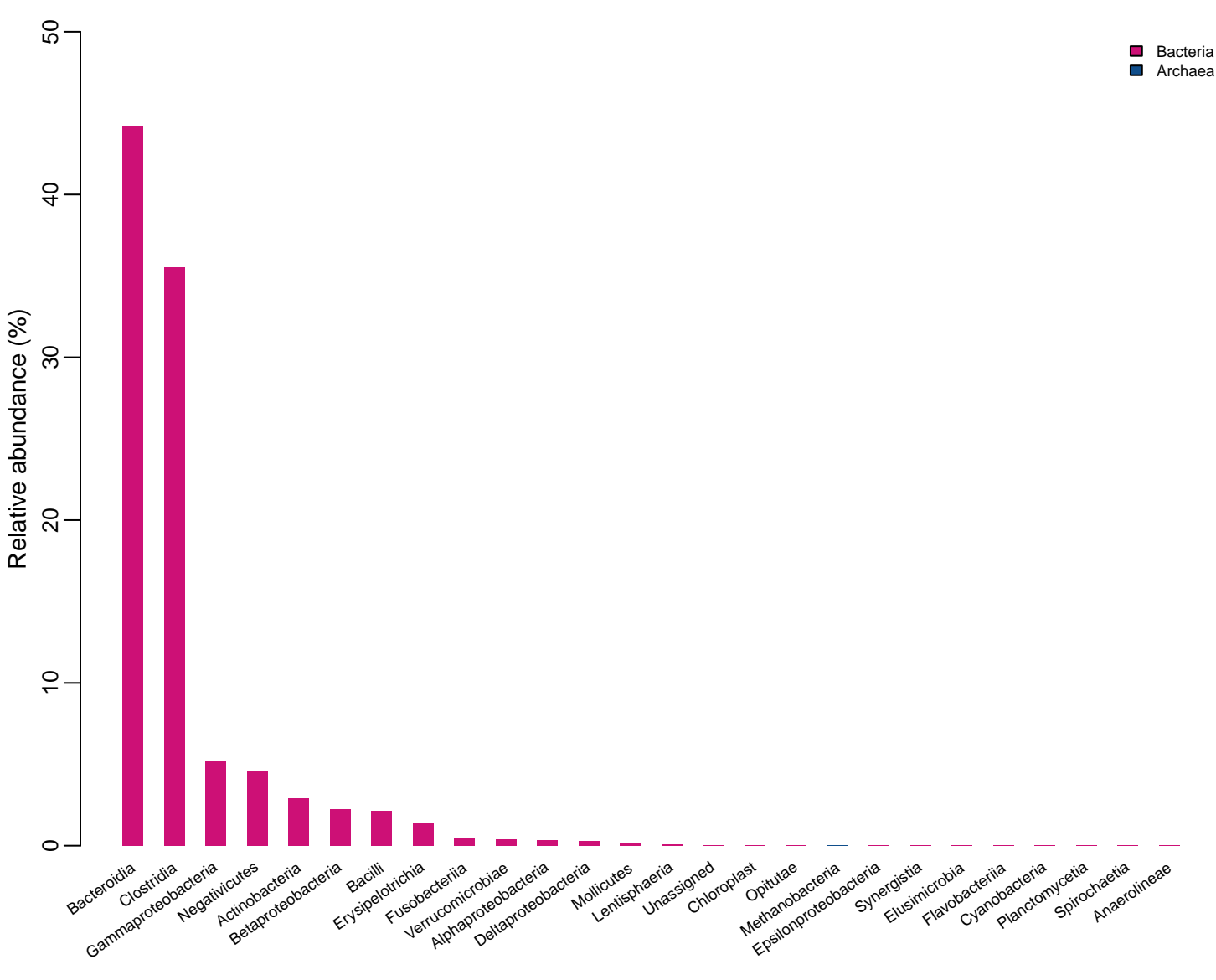

Supplement: Supplemental Information 1 [file peerj-09-10952-s001.zip › data/group_result_1_2_3/Community_Structure/class/class.taxon.Barplot.Legend.With.superkingdom.pdf]

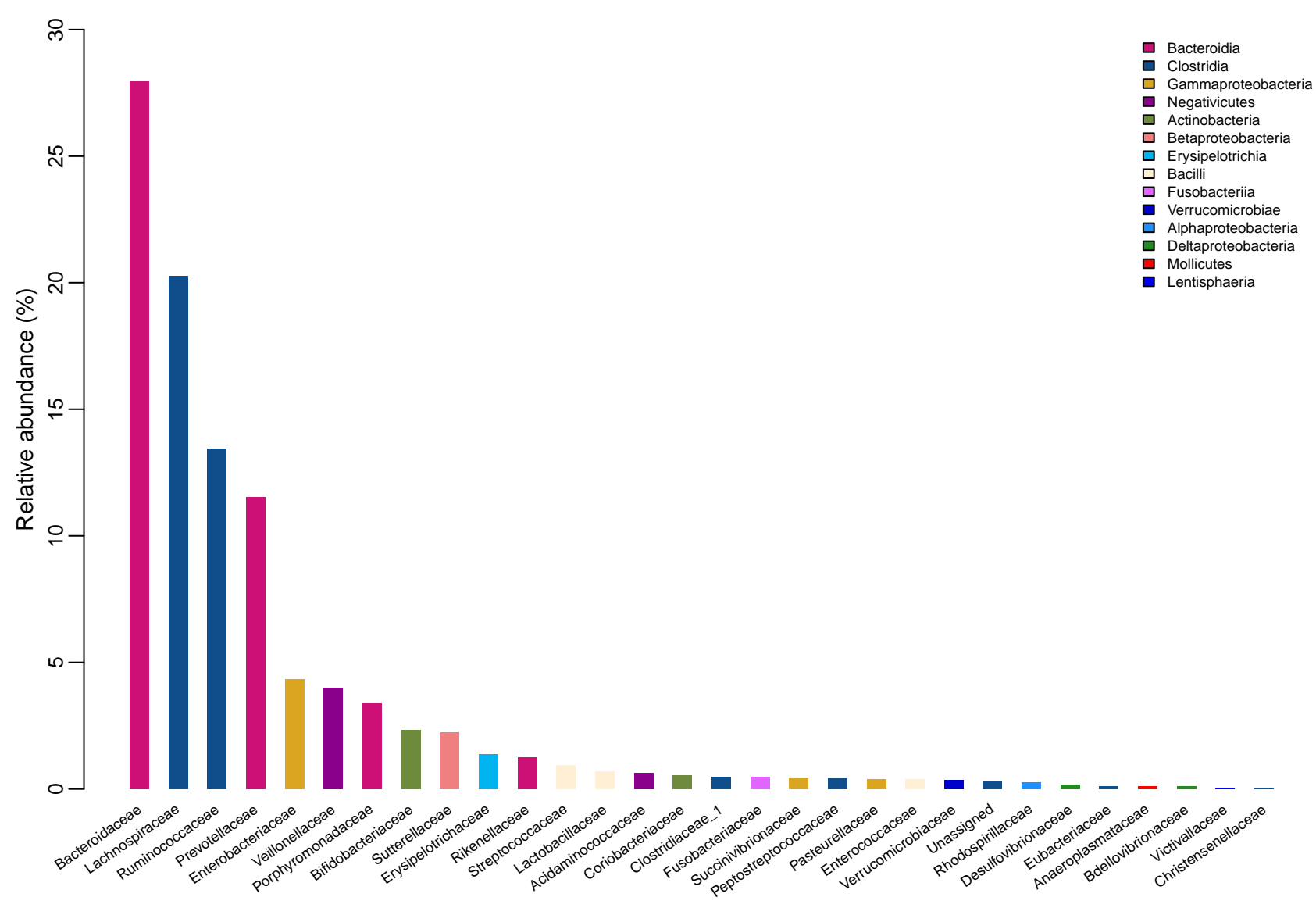

Supplement: Supplemental Information 1 [file peerj-09-10952-s001.zip › data/group_result_1_2_3/Community_Structure/family/family.taxon.Barplot.Legend.With.class.pdf]

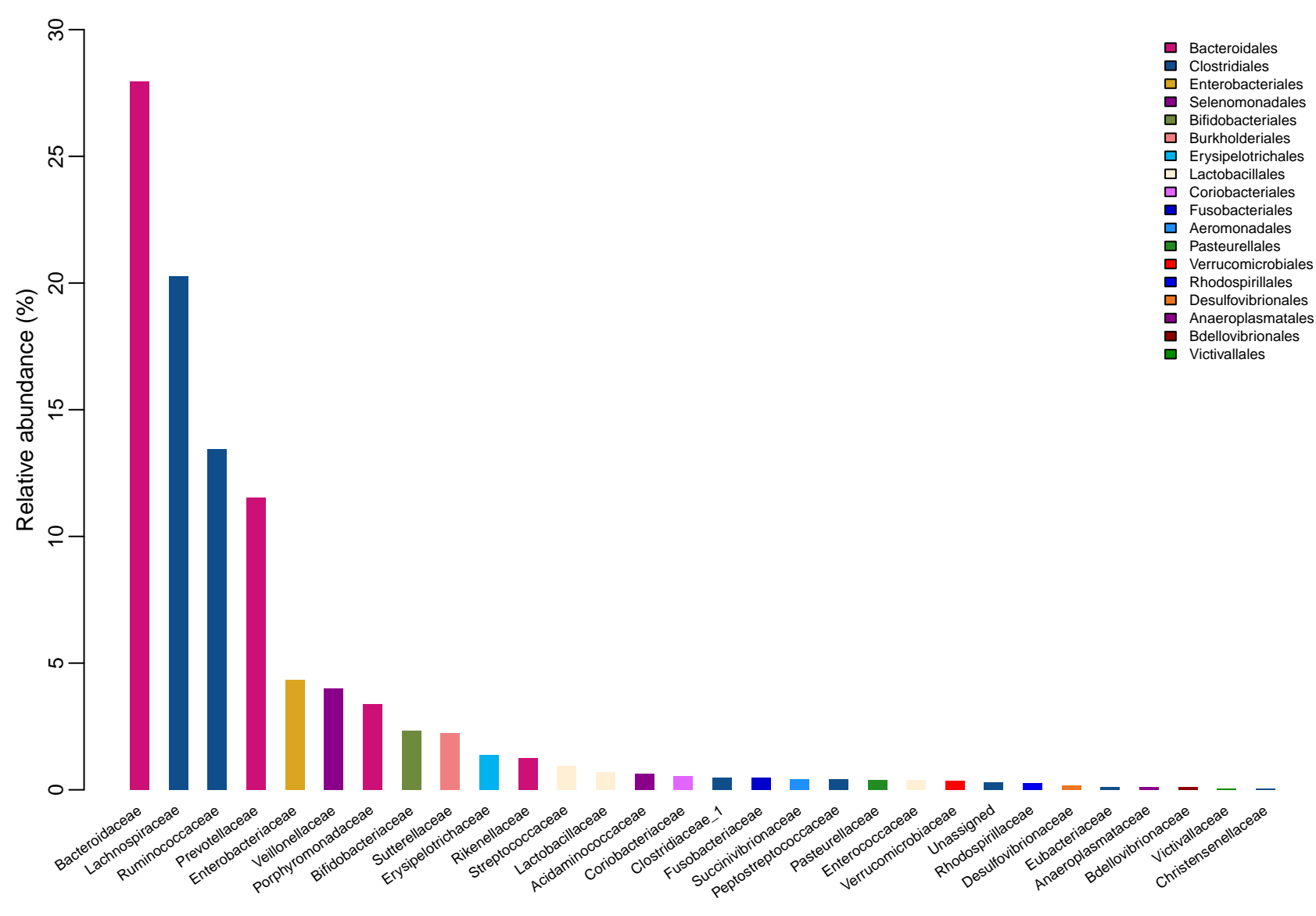

Supplement: Supplemental Information 1 [file peerj-09-10952-s001.zip › data/group_result_1_2_3/Community_Structure/family/family.taxon.Barplot.Legend.With.order.pdf]

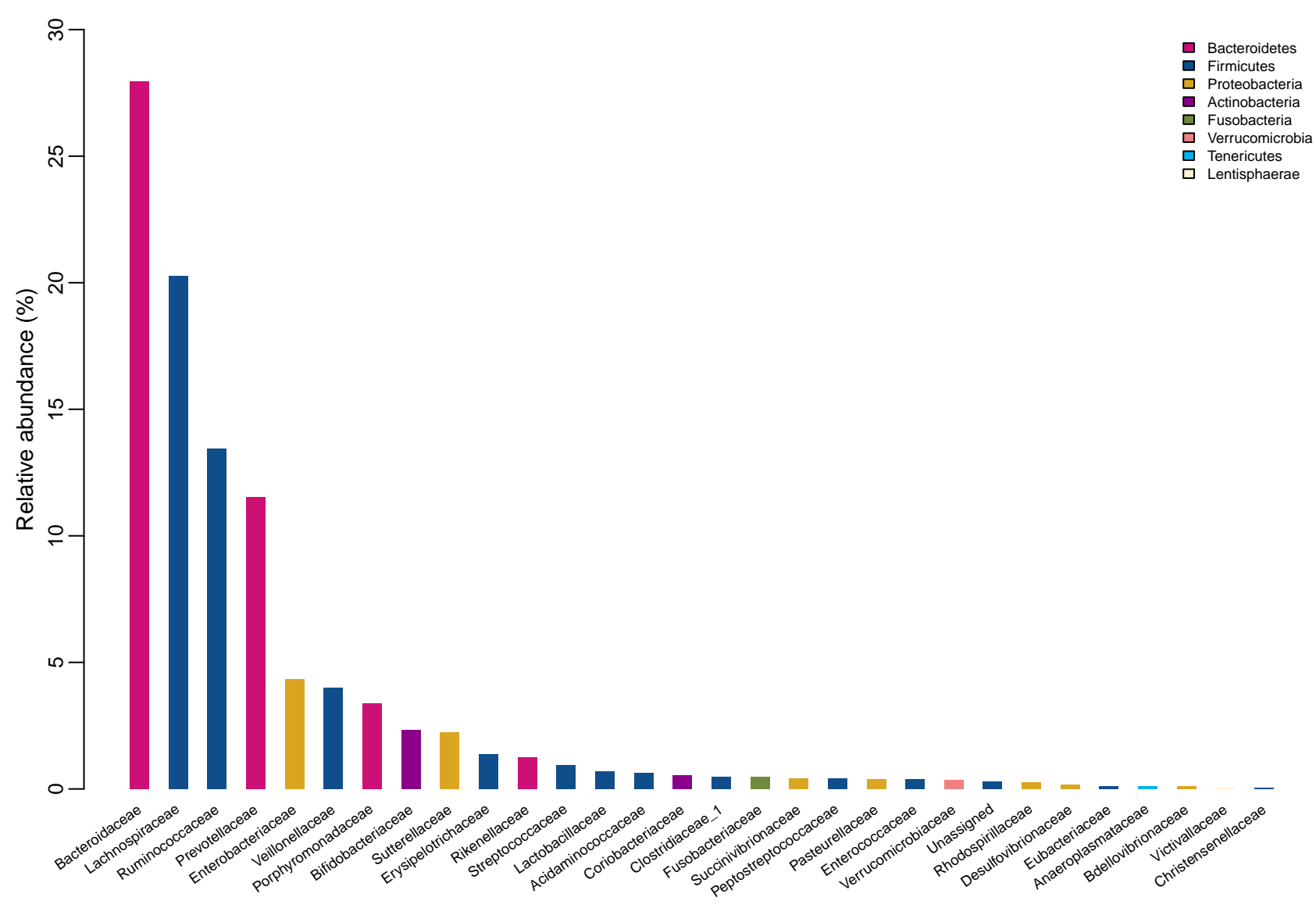

Supplement: Supplemental Information 1 [file peerj-09-10952-s001.zip › data/group_result_1_2_3/Community_Structure/family/family.taxon.Barplot.Legend.With.phylum.pdf]

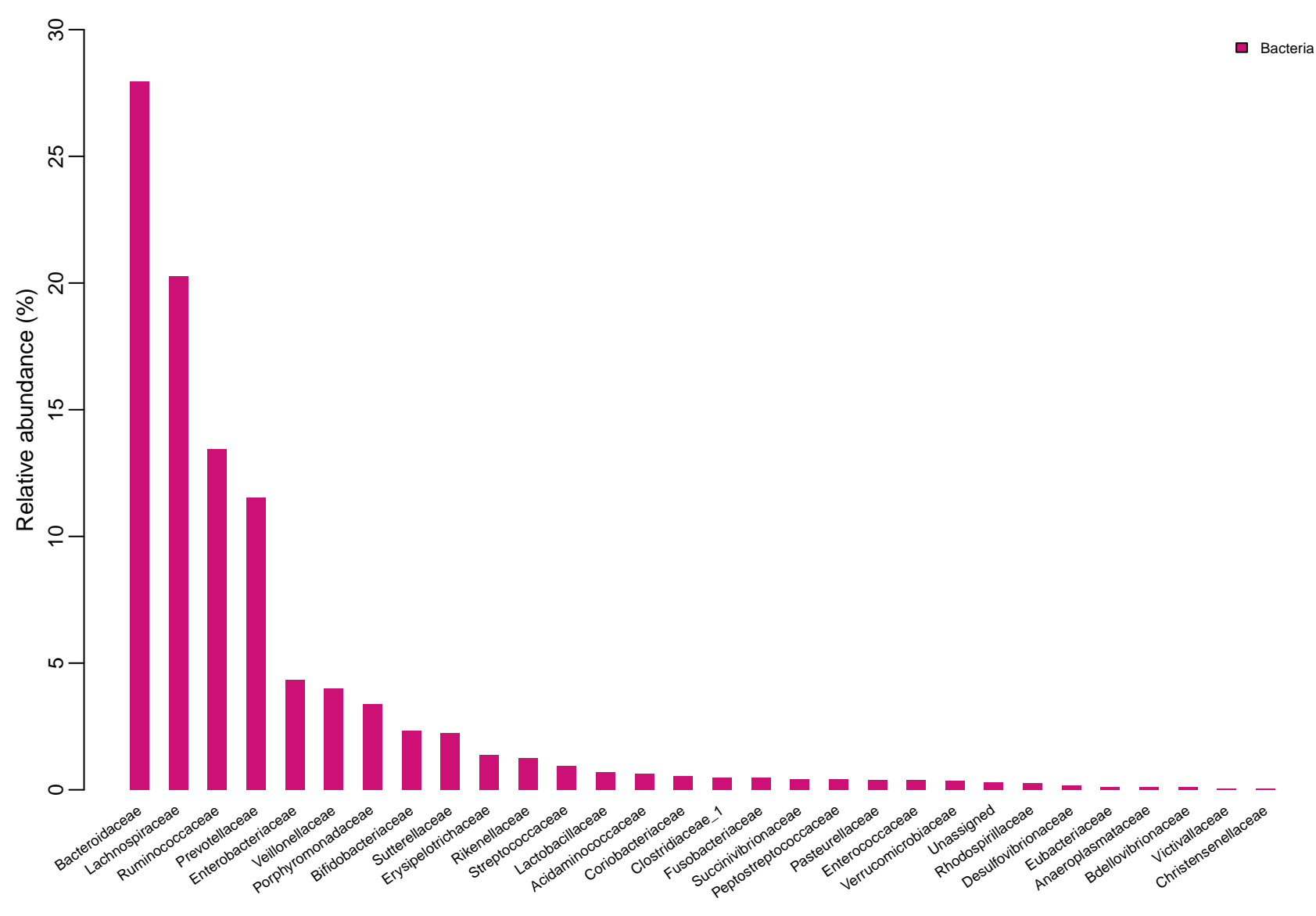

Supplement: Supplemental Information 1 [file peerj-09-10952-s001.zip › data/group_result_1_2_3/Community_Structure/family/family.taxon.Barplot.Legend.With.superkingdom.pdf]

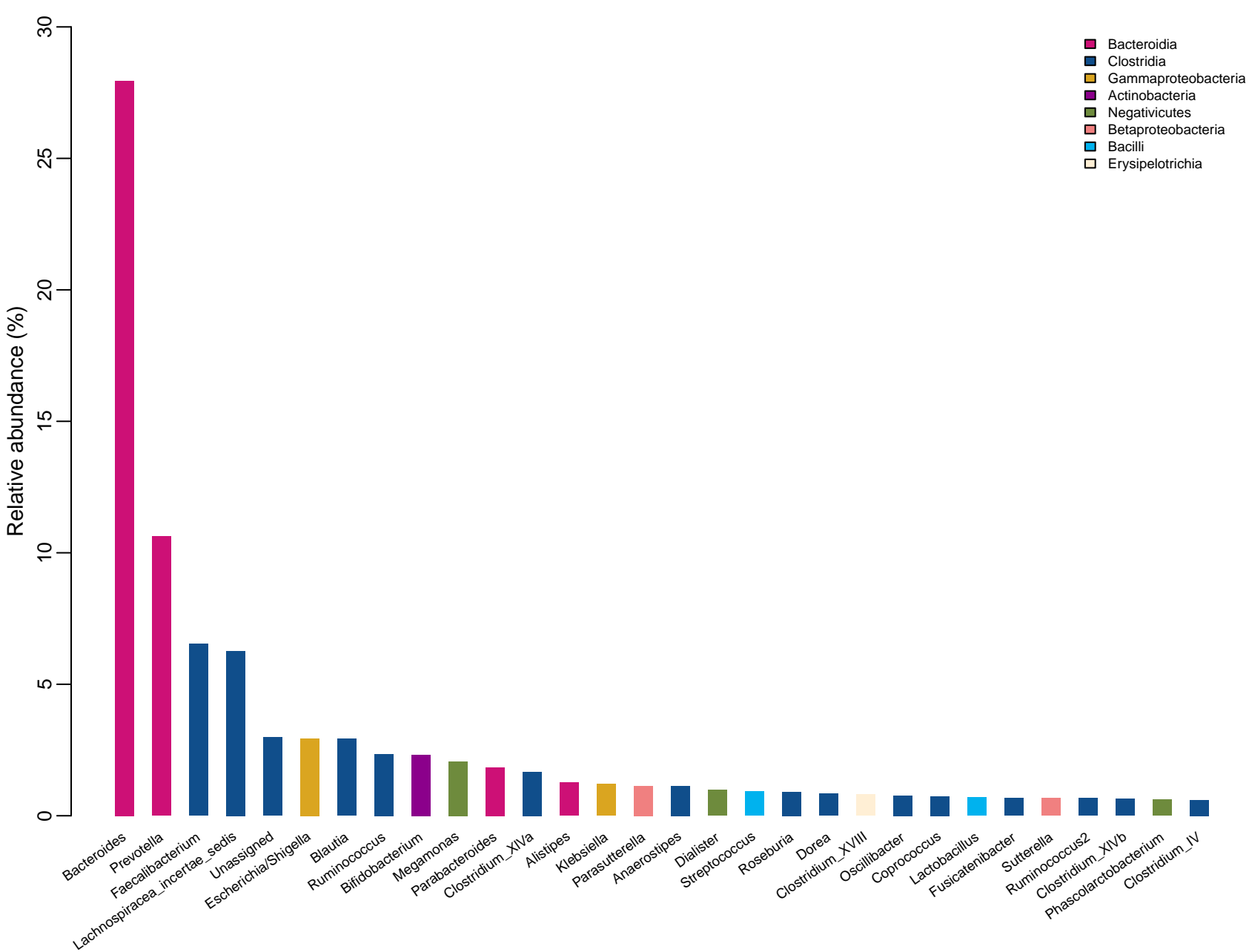

Supplement: Supplemental Information 1 [file peerj-09-10952-s001.zip › data/group_result_1_2_3/Community_Structure/genus/genus.taxon.Barplot.Legend.With.class.pdf]

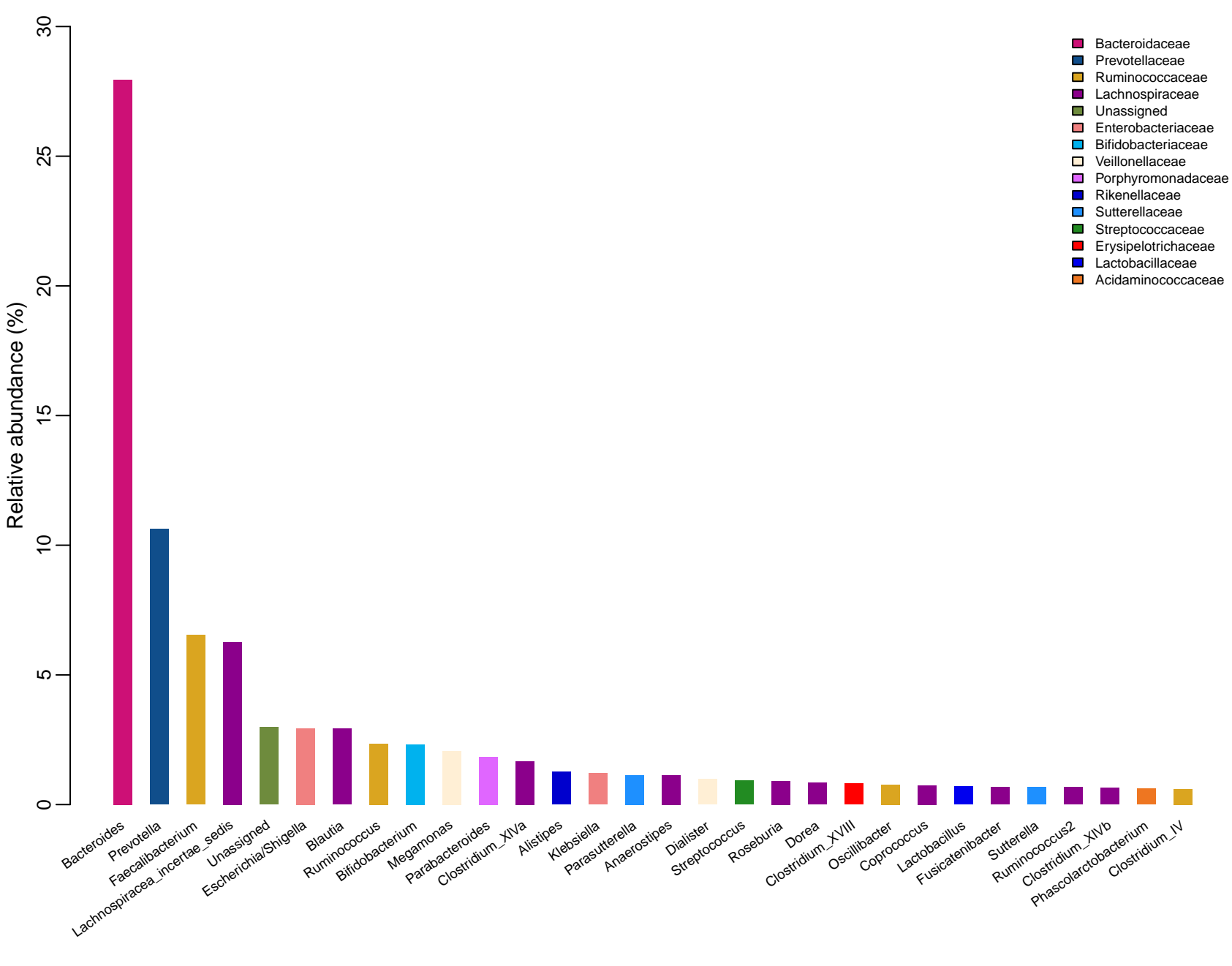

Supplement: Supplemental Information 1 [file peerj-09-10952-s001.zip › data/group_result_1_2_3/Community_Structure/genus/genus.taxon.Barplot.Legend.With.family.pdf]

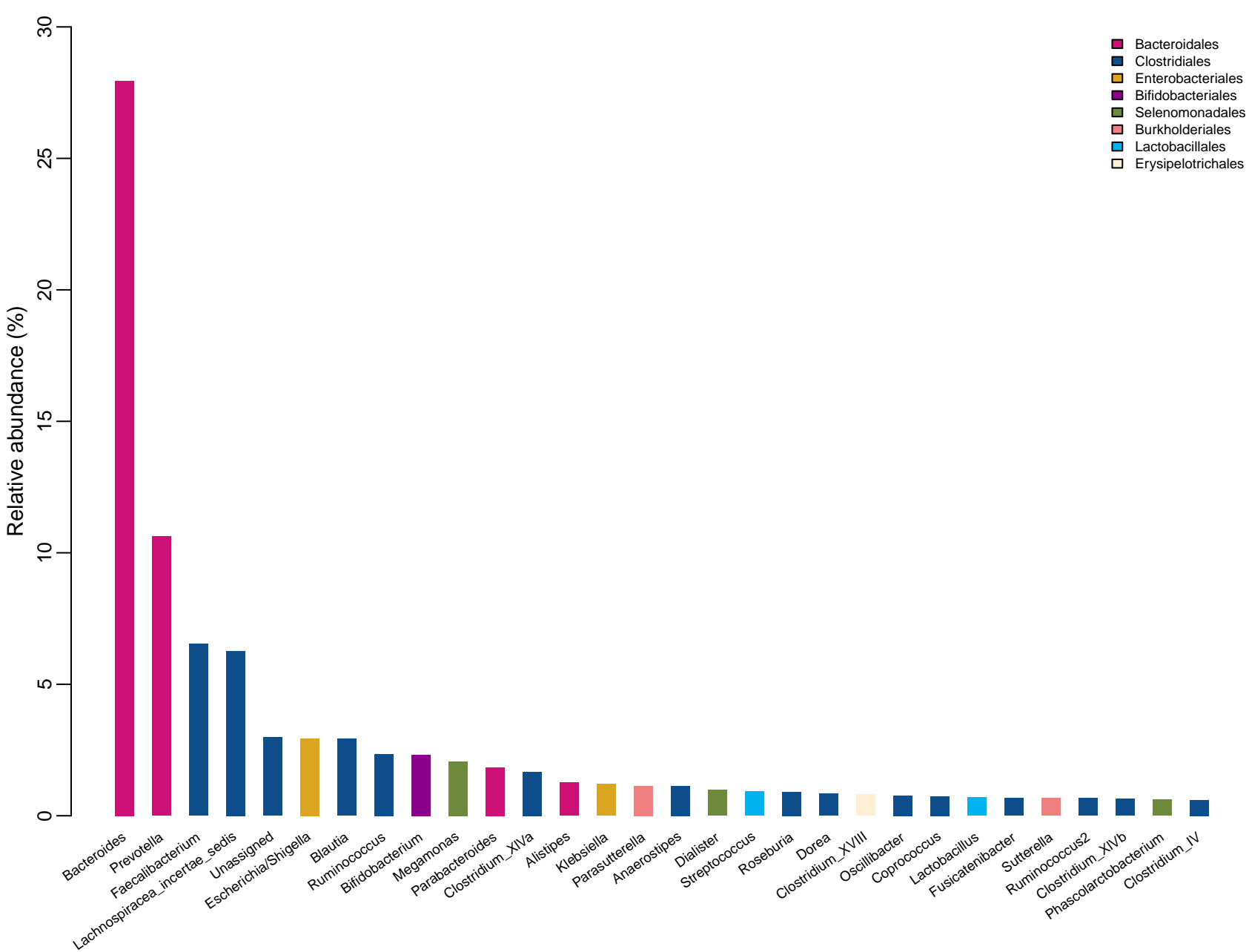

Supplement: Supplemental Information 1 [file peerj-09-10952-s001.zip › data/group_result_1_2_3/Community_Structure/genus/genus.taxon.Barplot.Legend.With.order.pdf]

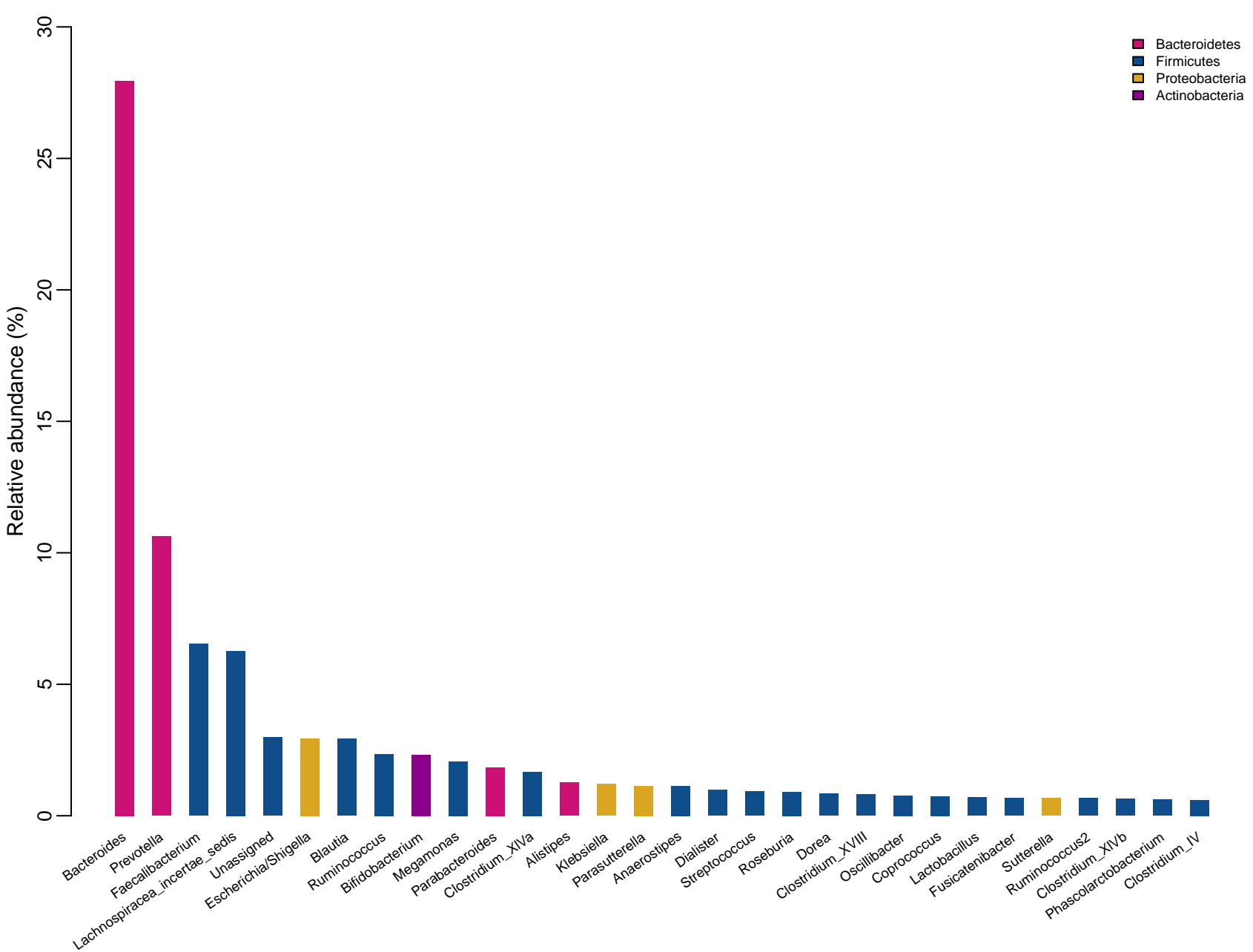

Supplement: Supplemental Information 1 [file peerj-09-10952-s001.zip › data/group_result_1_2_3/Community_Structure/genus/genus.taxon.Barplot.Legend.With.phylum.pdf]

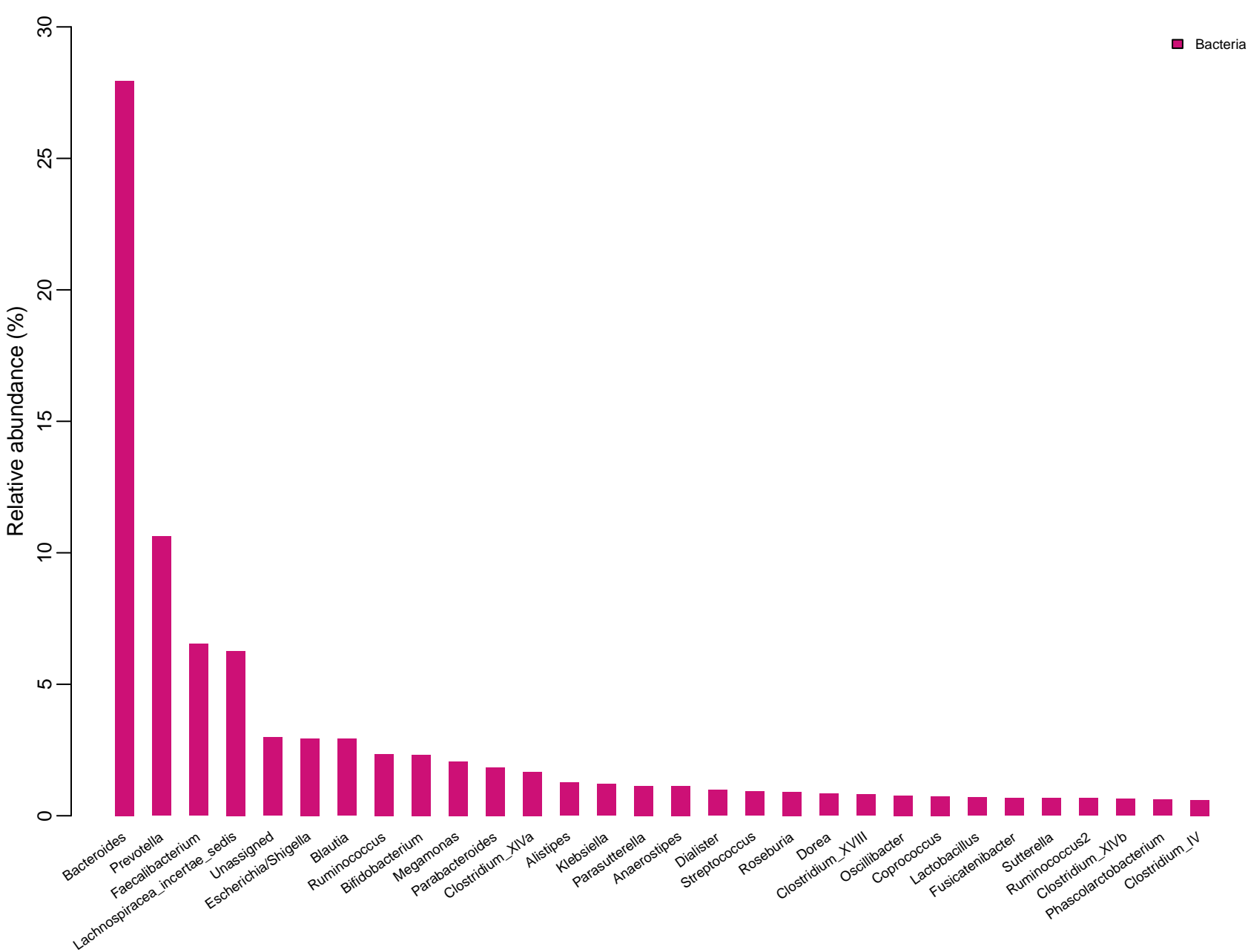

Supplement: Supplemental Information 1 [file peerj-09-10952-s001.zip › data/group_result_1_2_3/Community_Structure/genus/genus.taxon.Barplot.Legend.With.superkingdom.pdf]

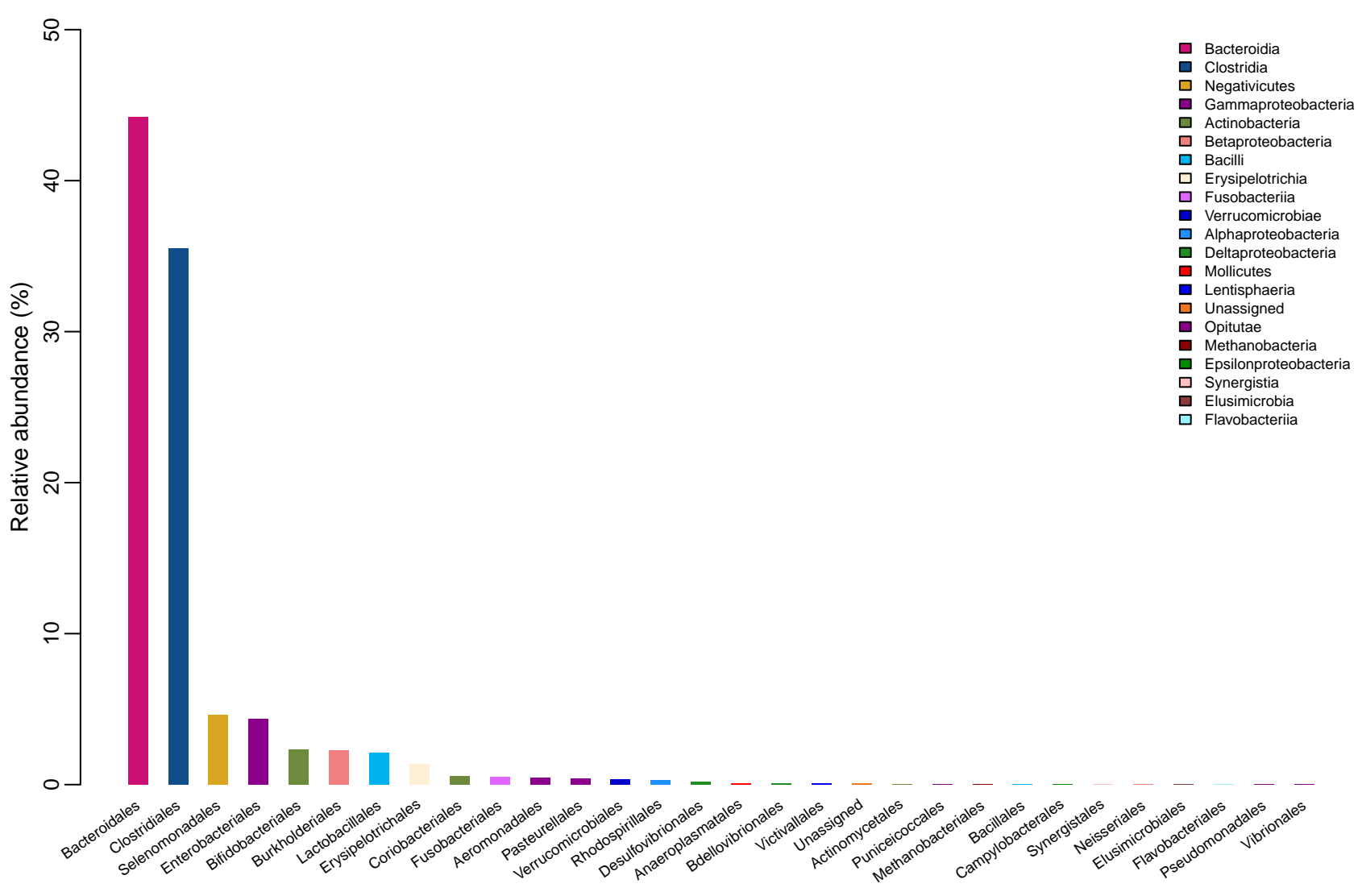

Supplement: Supplemental Information 1 [file peerj-09-10952-s001.zip › data/group_result_1_2_3/Community_Structure/order/order.taxon.Barplot.Legend.With.class.pdf]

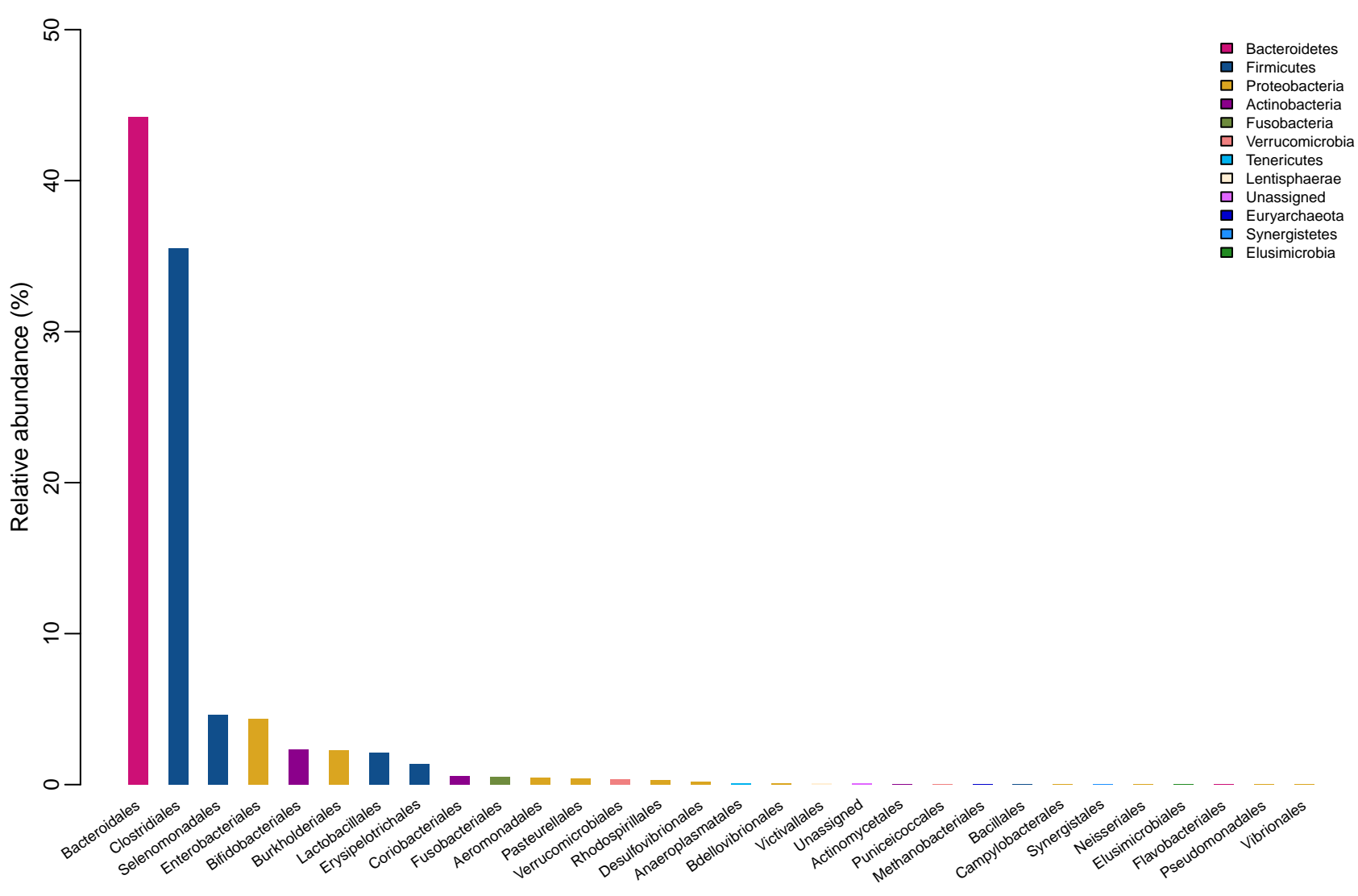

Supplement: Supplemental Information 1 [file peerj-09-10952-s001.zip › data/group_result_1_2_3/Community_Structure/order/order.taxon.Barplot.Legend.With.phylum.pdf]

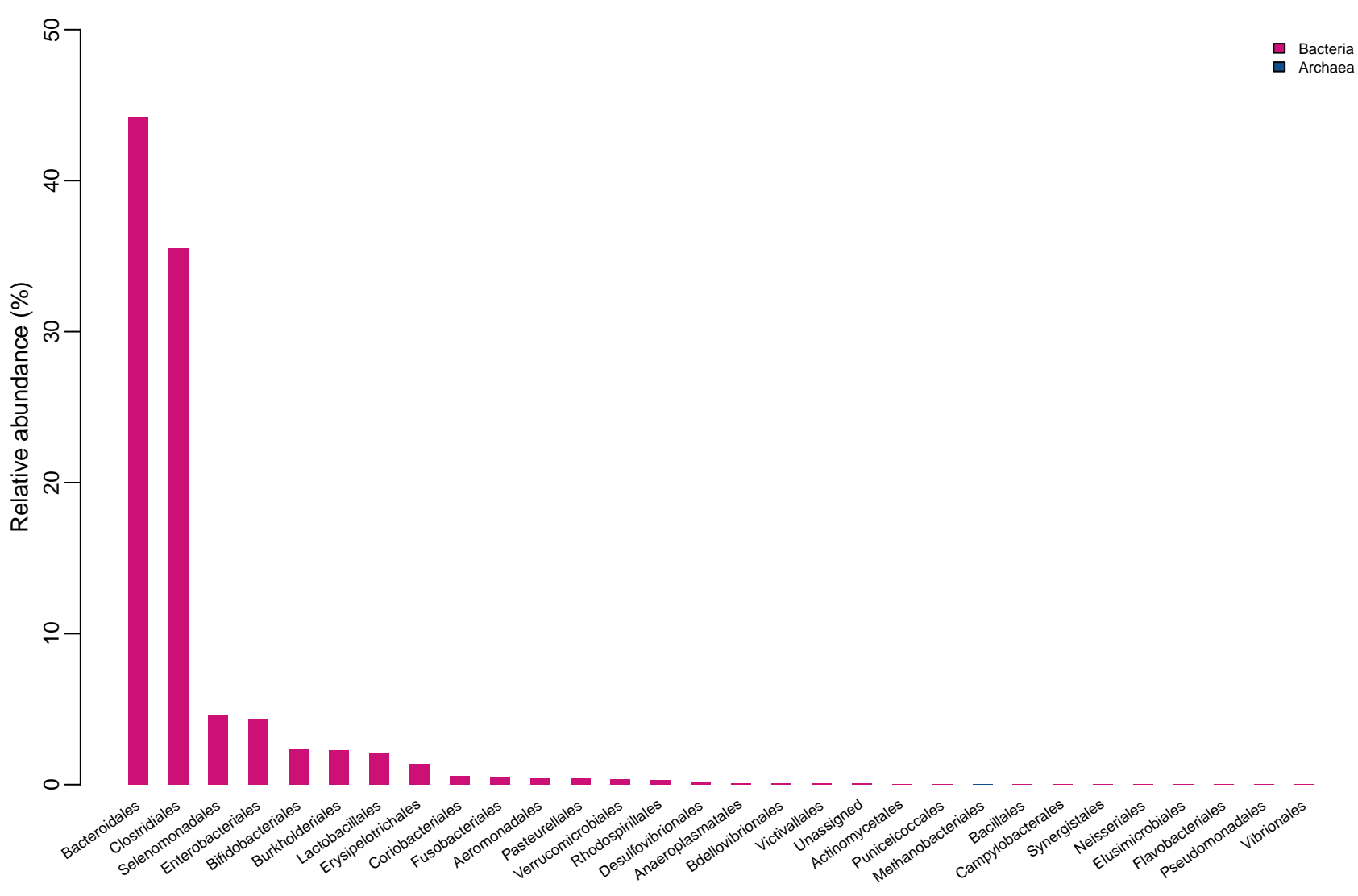

Supplement: Supplemental Information 1 [file peerj-09-10952-s001.zip › data/group_result_1_2_3/Community_Structure/order/order.taxon.Barplot.Legend.With.superkingdom.pdf]

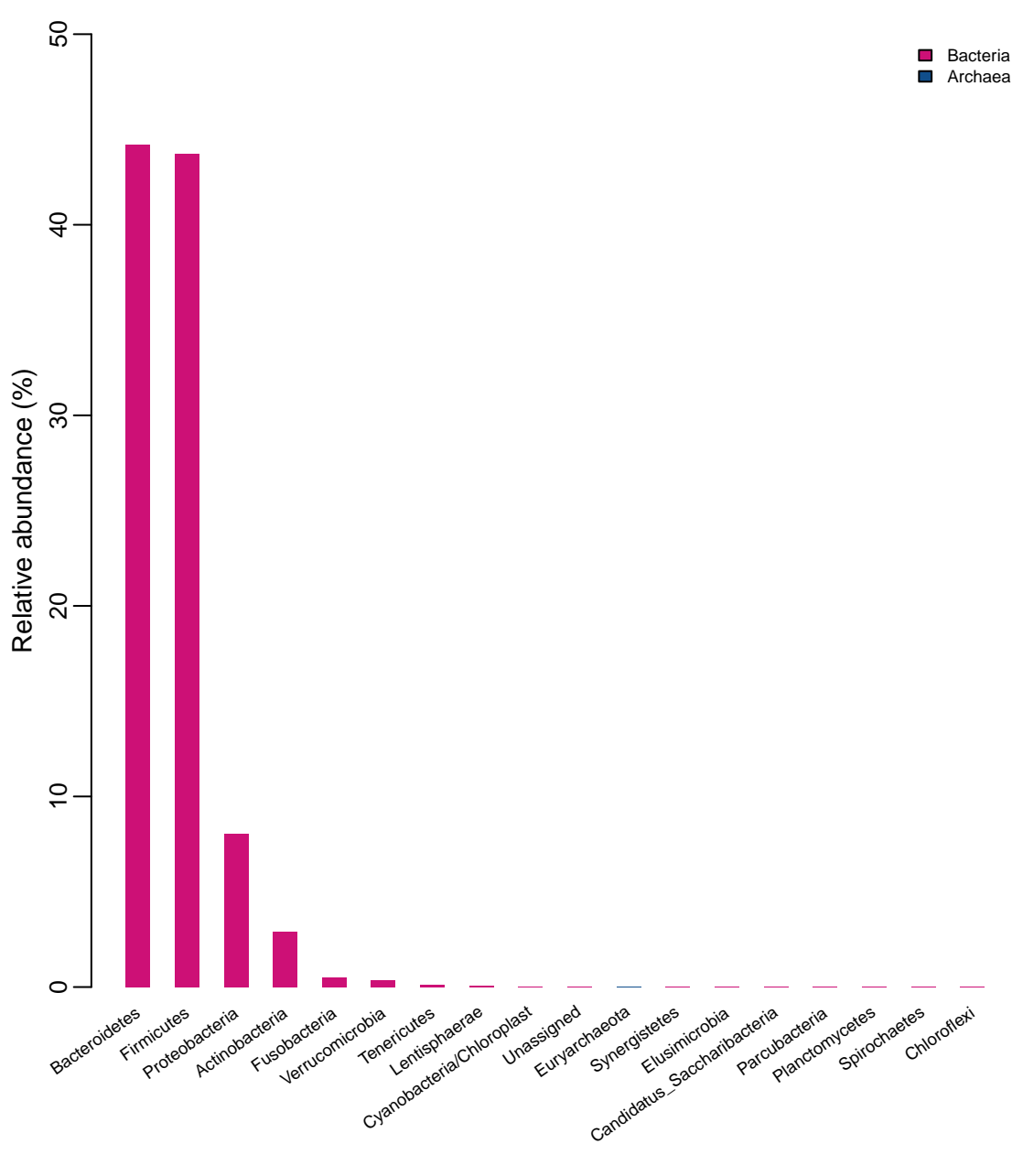

Supplement: Supplemental Information 1 [file peerj-09-10952-s001.zip › data/group_result_1_2_3/Community_Structure/phylum/phylum.taxon.Barplot.Legend.With.superkingdom.pdf]

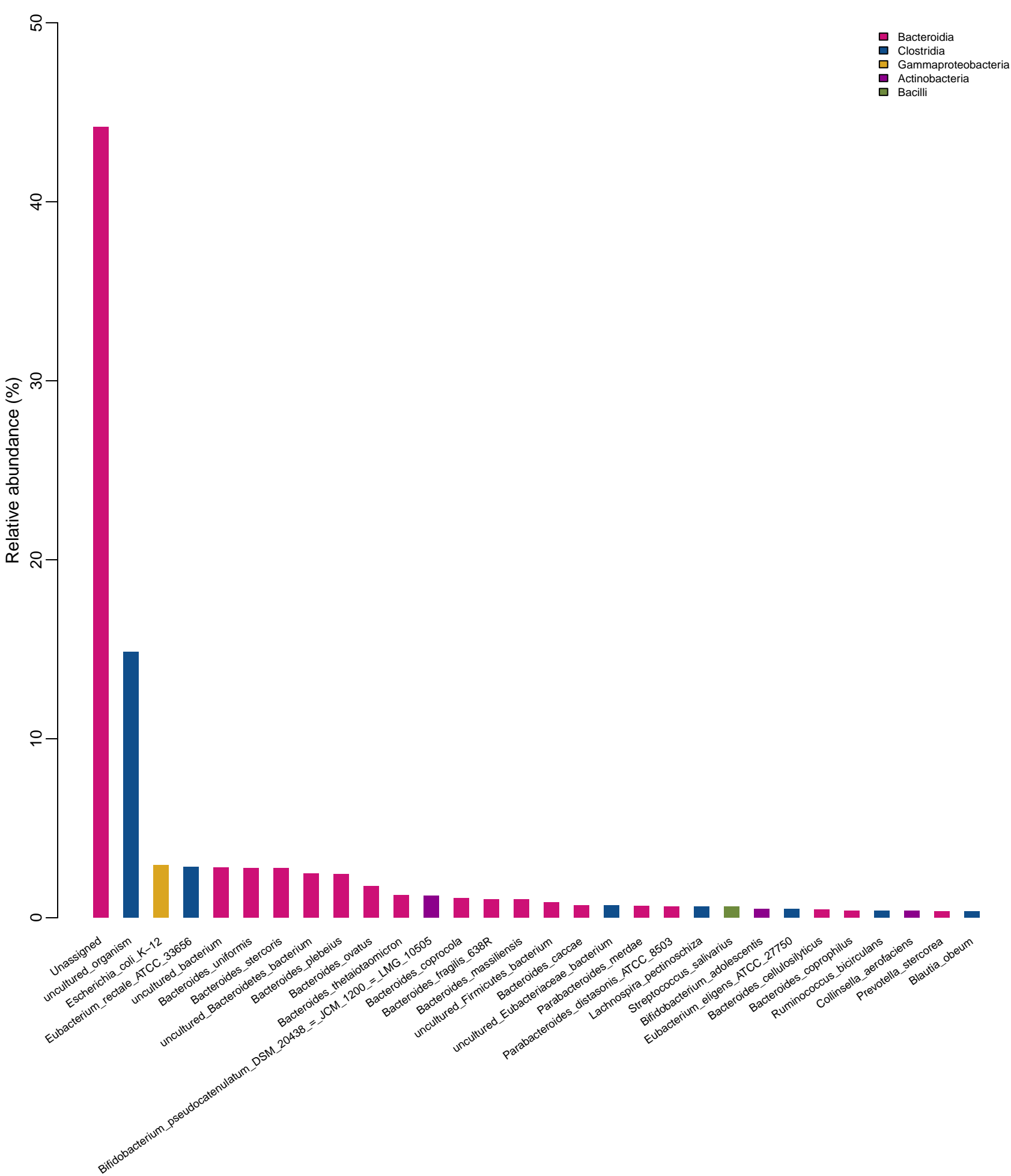

Supplement: Supplemental Information 1 [file peerj-09-10952-s001.zip › data/group_result_1_2_3/Community_Structure/species/species.taxon.Barplot.Legend.With.class.pdf]

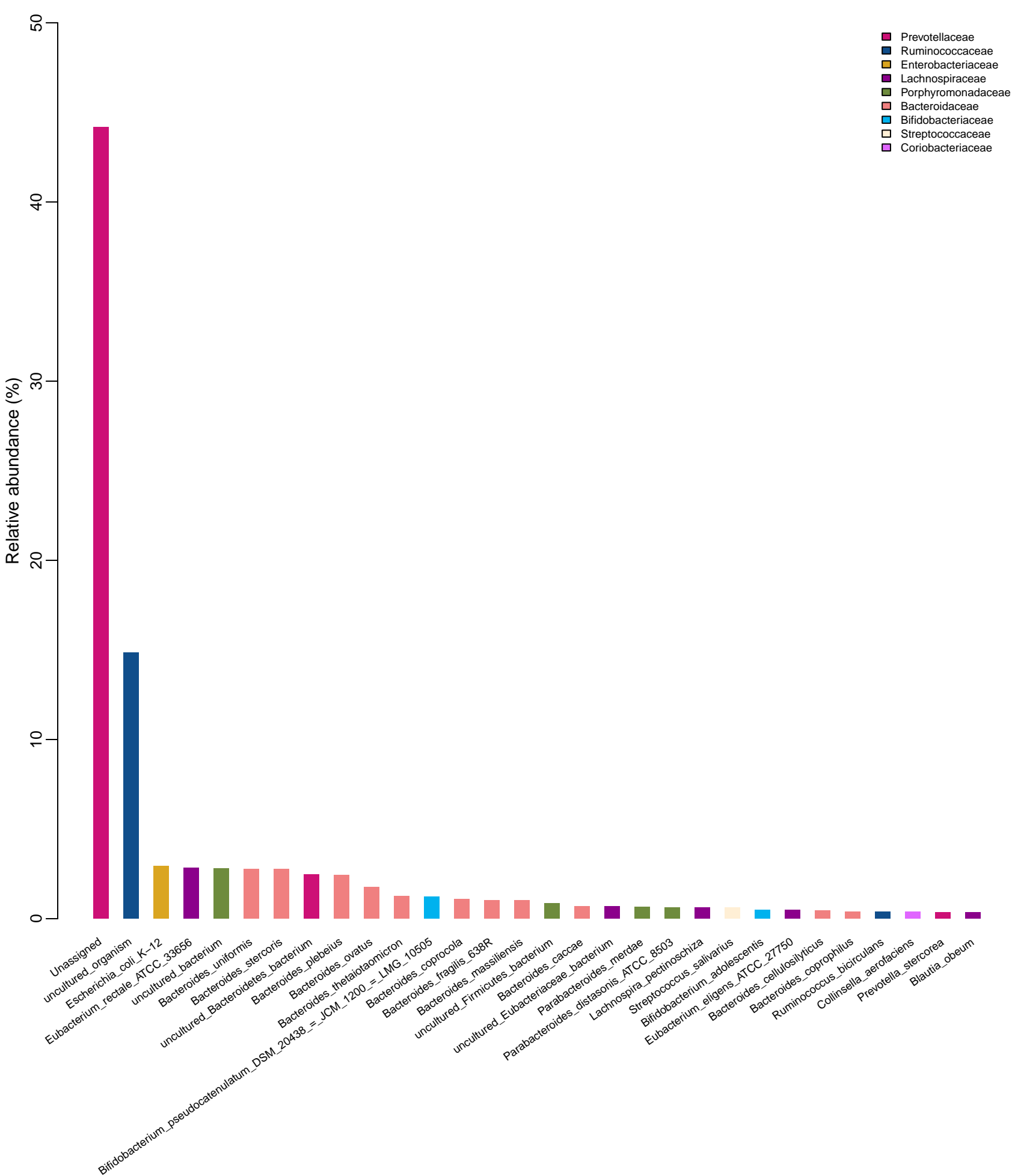

Supplement: Supplemental Information 1 [file peerj-09-10952-s001.zip › data/group_result_1_2_3/Community_Structure/species/species.taxon.Barplot.Legend.With.family.pdf]

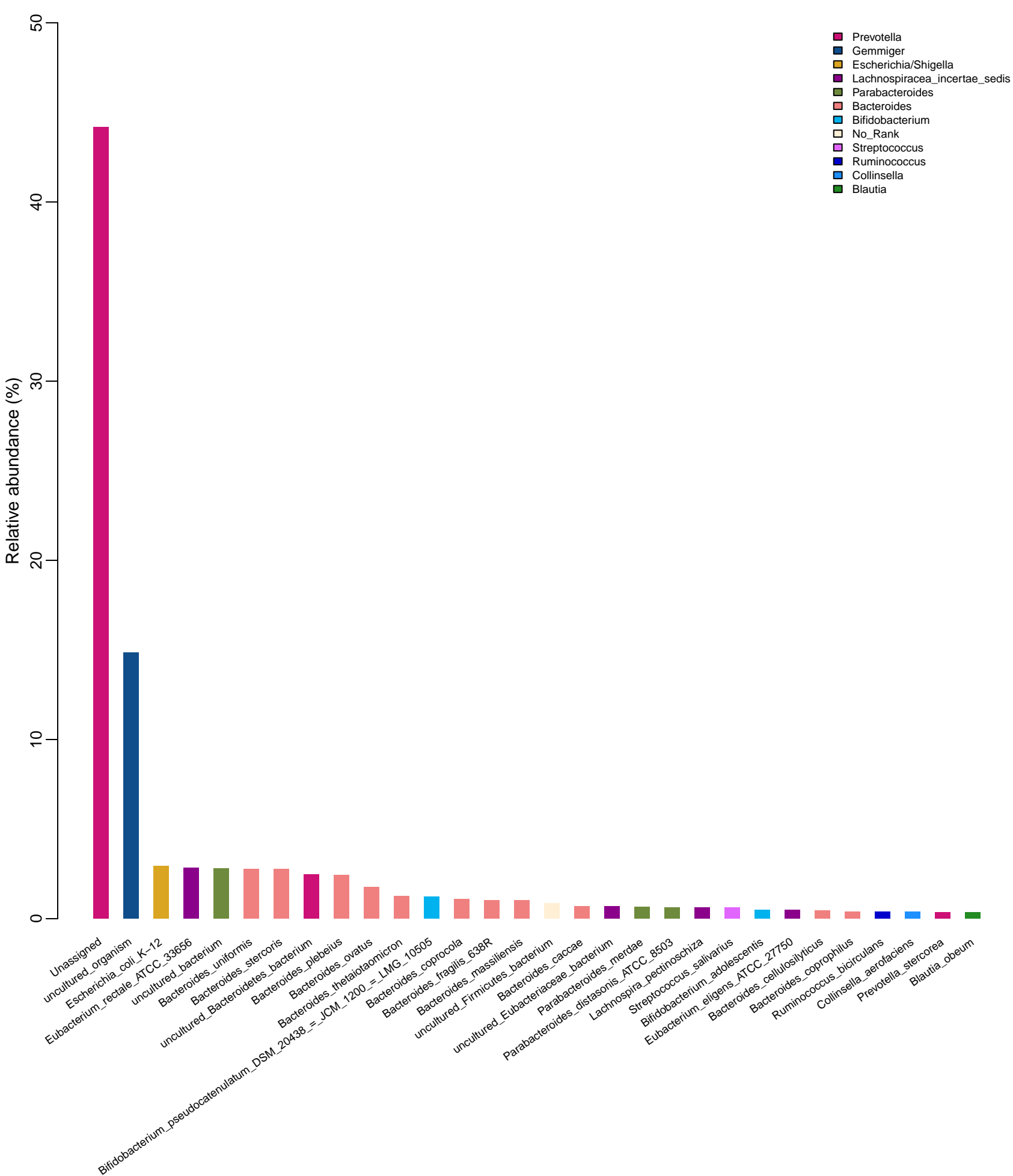

Supplement: Supplemental Information 1 [file peerj-09-10952-s001.zip › data/group_result_1_2_3/Community_Structure/species/species.taxon.Barplot.Legend.With.genus.pdf]

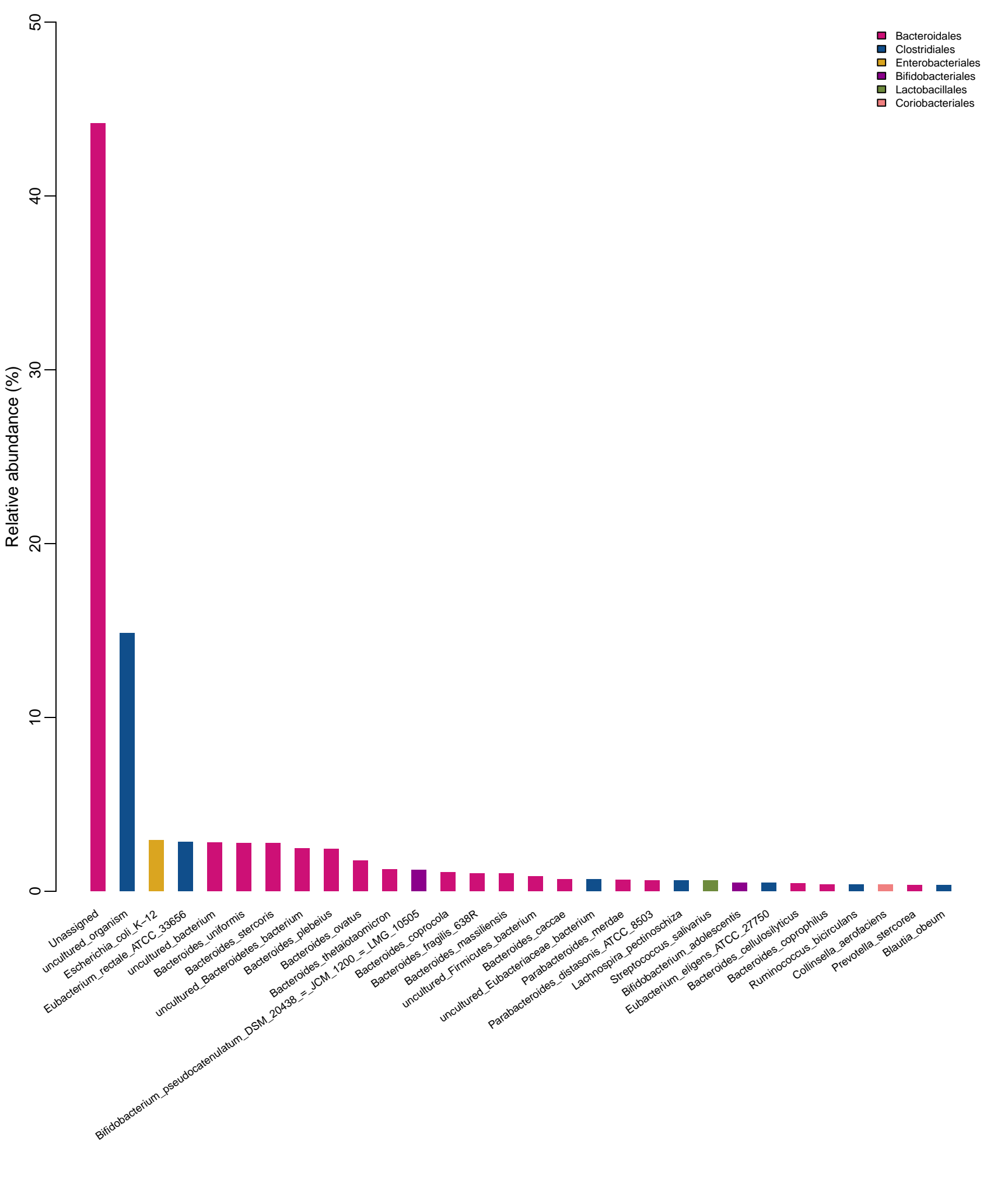

Supplement: Supplemental Information 1 [file peerj-09-10952-s001.zip › data/group_result_1_2_3/Community_Structure/species/species.taxon.Barplot.Legend.With.order.pdf]

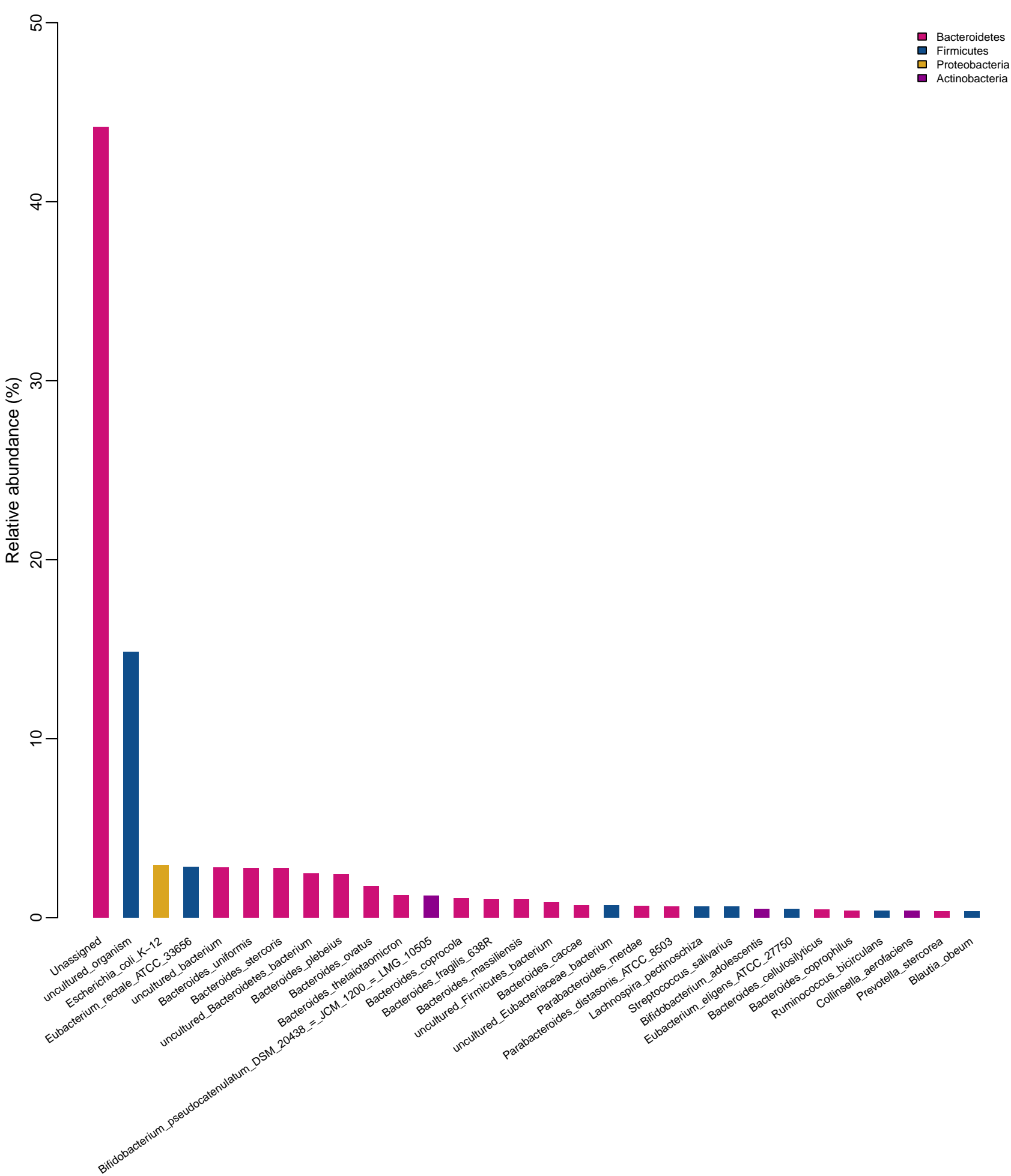

Supplement: Supplemental Information 1 [file peerj-09-10952-s001.zip › data/group_result_1_2_3/Community_Structure/species/species.taxon.Barplot.Legend.With.phylum.pdf]

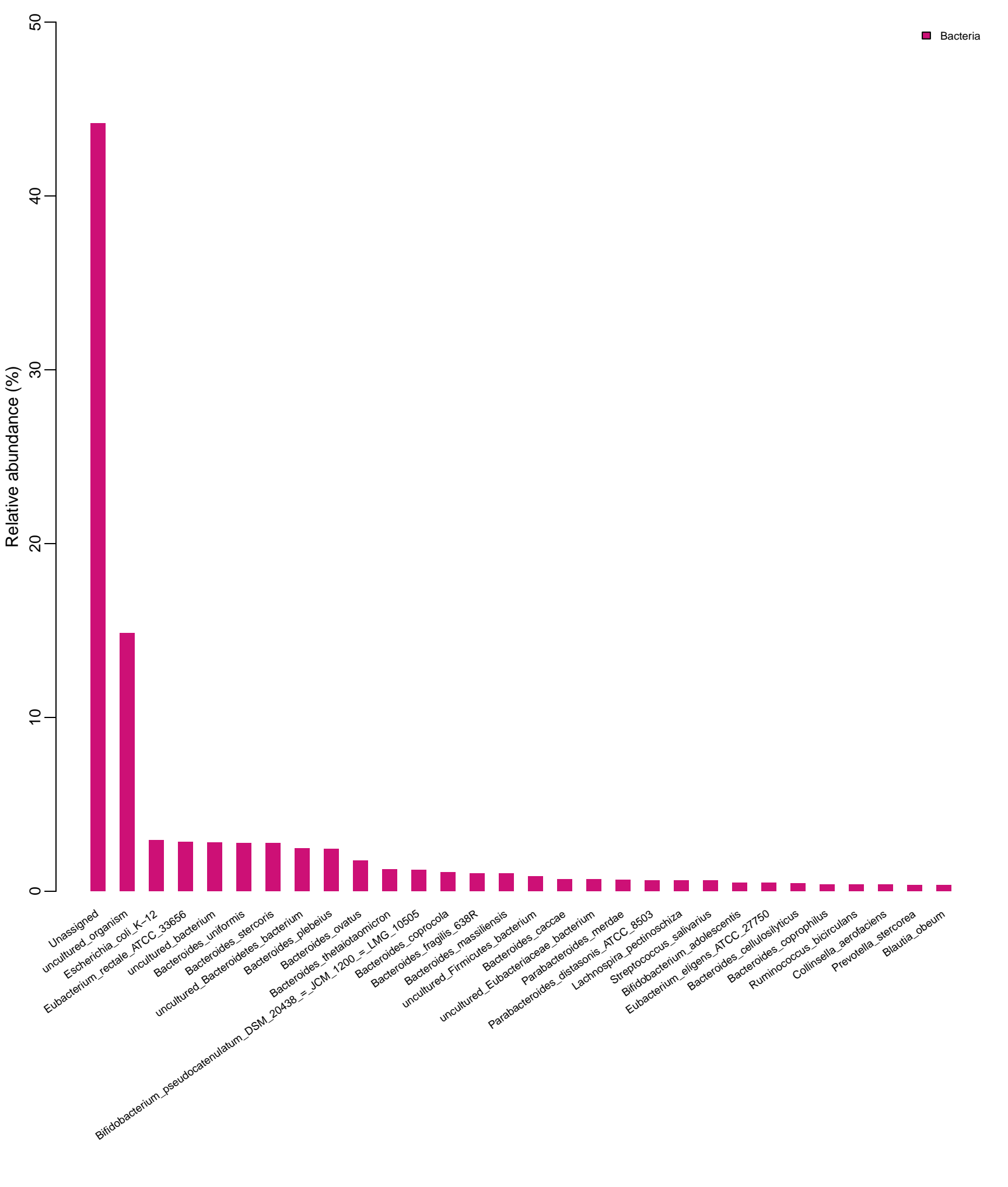

Supplement: Supplemental Information 1 [file peerj-09-10952-s001.zip › data/group_result_1_2_3/Community_Structure/species/species.taxon.Barplot.Legend.With.superkingdom.pdf]

Sequence length distribution

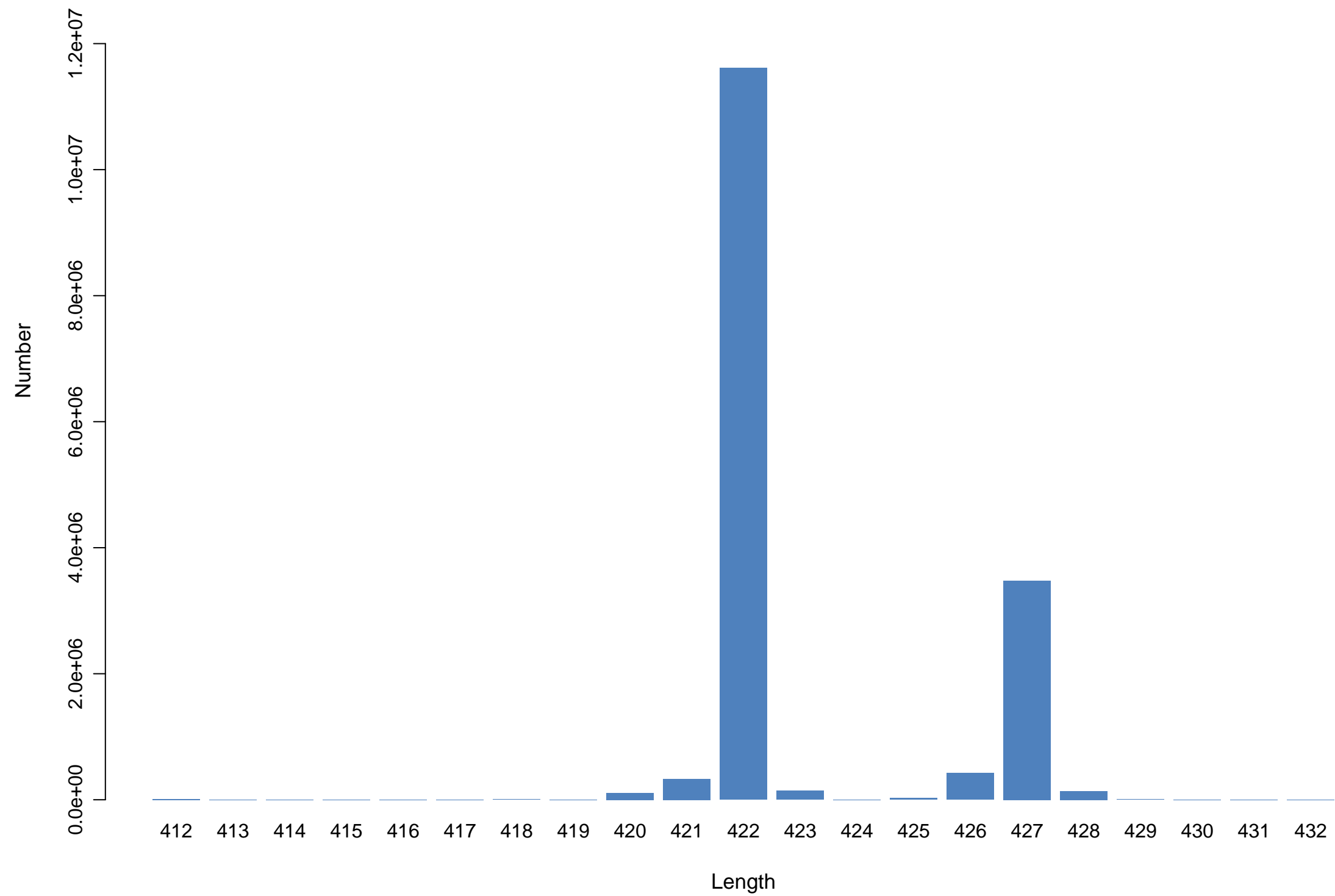

Supplement: Supplemental Information 2 [file peerj-09-10952-s002.zip › sequence/Statistics/reads.clean.length.distribution.pdf]

# Samples Rarefaction Curves

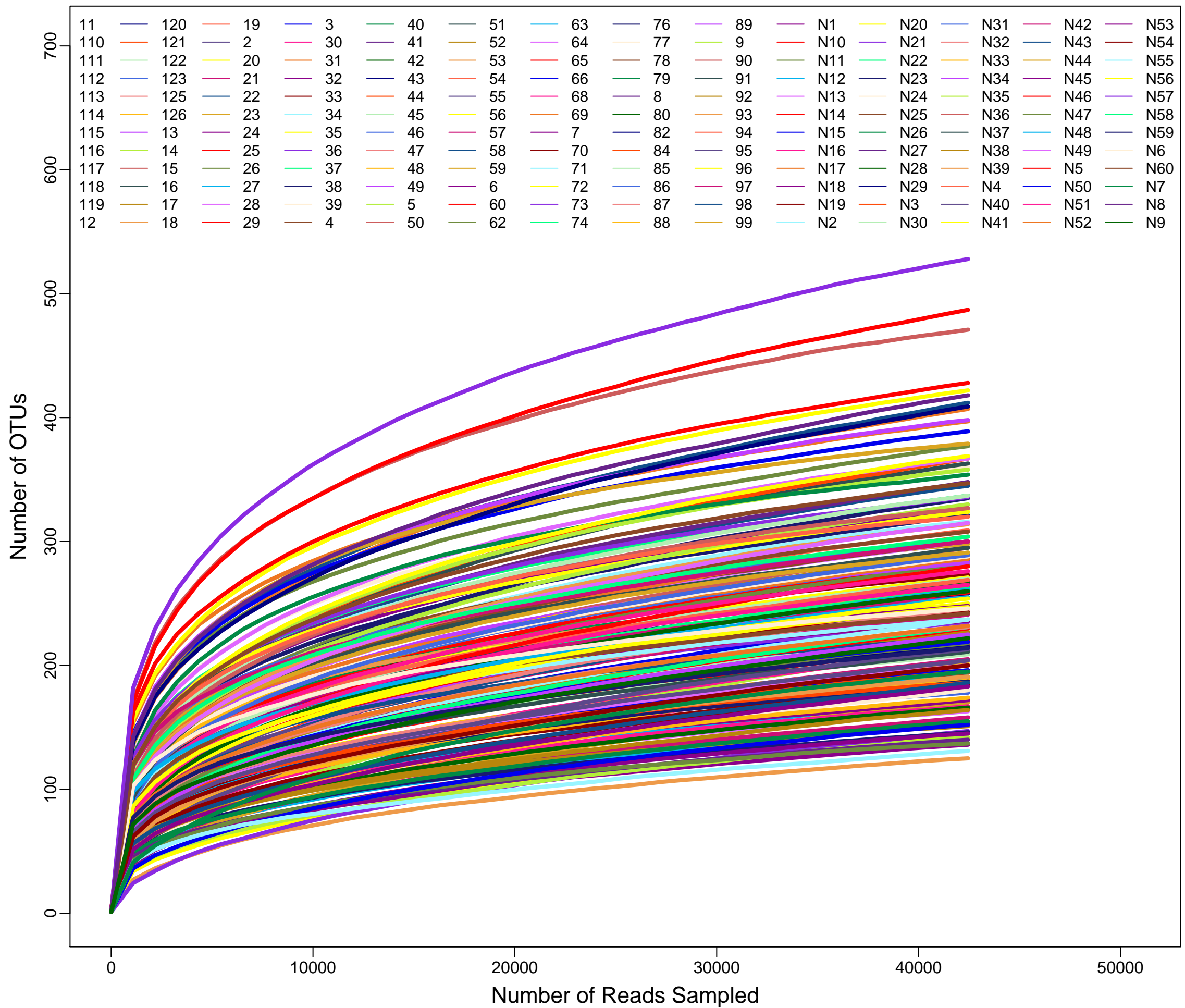

Supplement: Figure S1 [file peerj-09-10952-s004.pdf]
