# Supplementary material for: Characteristics of the gut microbiome in patients with prediabetes and type 2 diabetes
Source: PeerJ. 2021 Mar 24;9:e10952. doi: 10.7717/peerj.10952 (PMC8000457; doi:10.7717/peerj.10952)
Supplement: Supplemental Information 3 [file peerj-09-10952-s003.docx]

| variable | code |
| --- | --- |
| Group | 1=T2DM,2=preDM,3=Control |
| Gender | 1=Men,2=Women |
| Education | 1=Without formal education,2=Primary, 3=Middle school, 4=High school and above |
| Annual income(yuan) | 1=≤9,999, 2=10,000-19,999, 3=≥20,000 |
| Cat_drink | 1=yes, 0=NO |
| Cat_smoke | 1=yes, 0=NO |
| Diet | 1=≥1～3times/week，2=little or not |
