# Supplementary material for: Characteristics of the gut microbiome in patients with prediabetes and type 2 diabetes
Source: PeerJ. 2021 Mar 24;9:e10952. doi: 10.7717/peerj.10952 (PMC8000457; doi:10.7717/peerj.10952)
Supplement: Table S1 [file peerj-09-10952-s005.docx]

Diabetes-associated differences in gut microbiome abundance: comparison with previous studies

| [Category](#/javascript:;) | Previous studies | T2DM compare to healthy controls | Our study |
| --- | --- | --- | --- |
| *Firmicutes* | Navab-Moghada et al. 2017 | Higher | No difference |
|  | Ahmad et al. 2019 | Higher |  |
|  | Palacios et al. 2017 | Lower |  |
|  | Lambeth et al. 2015 | No difference |  |
| *Bacteroidetes* | Navab-Moghada et al. 2017 | lower | No difference |
|  | Ahmad et al. 2019 | lower |  |
|  | Palacios et al. 2017 | Higher |  |
| *Lactobacillus* | Palacios et al. 2017 | Lower | Higher in T2DM;  Lower in preDM |
|  | Sedighi et al. 2017 | Higher |  |
| *Bifidobacteria* | Sedighi et al. 2017 | Lower | No difference |
|  | Palacios et al. 2017 | Lower |  |
| *Proteobacteria* | Larsen et al. 2010 | Higher | Higher in preDM |
| *Prevotella* | Zhang et al. 2013 | Higher | Higher |
|  | Ahmad et al. 2019 | Higher |  |
|  | Gaike A H et al.2020 | Lower |  |
| Megasphaera | Gaike A H et al.2020 | Higher | Higher |
