# Supplementary material for: Characteristics of the gut microbiome in patients with prediabetes and type 2 diabetes
Source: PeerJ. 2021 Mar 24;9:e10952. doi: 10.7717/peerj.10952 (PMC8000457; doi:10.7717/peerj.10952)
Supplement: Files S1 [file peerj-09-10952-s006.docx]

In order to obtain high quality sequencing data to improve the accuracy of subsequent analysis, quality control and filtering of the raw offline data is performed with the following main steps.

a. Removal of bases with an end mass of less than 20 and removal of possible adapter sequences using TrimGalore software, followed by removal of short sequences less than 100 bp in length.

b. Splicing of paired sequences obtained by double-end sequencing using FLASH2 software to obtain merge sequences, with further removal of post-merge low quality sequences (more than 90% of bases below 20).

c. find and remove primers from the sequence using mothur software.

d. Use usearch to remove sequences with a total base error rate greater than 2 and sequences less than 100 bp in length to obtain optimized sequences (Clean reads) of higher quality and confidence, which will be used for subsequent bioinformatic analysis.

Software used: TrimGalore, FLASH2, mother, usearch
